# A Qualitative Analysis of Organizational Practices to Support Peer Support Workers in The Substance Use Disorder Recovery Field

**DOI:** 10.1007/s11414-025-09980-0

**Published:** 2025-11-04

**Authors:** Sarah J. Miller, Sarah Grace Frary, Eleanor Wu, Wendy Chu, Magdalena Moskal, Elizabeth Bodalski, Sayward E. Harrison

**Affiliations:** 1 Department of Psychology, University of South Carolina, 1512 Pendleton St., Columbia, SC 29208 USA; 2 Department of Psychiatry and Behavioral Sciences, Johns Hopkins University, Baltimore, MD USA

## Abstract

Peer support workers (PSWs) play a key role in the substance use disorder (SUD) recovery field. According to organizational support theory, organizational actions and policies are key factors that promote perceived support and positive job-related outcomes. However, limited qualitative research has examined the construct of perceived organizational support from the perspective of PSWs working in the SUD recovery field. This study sought to determine what organizational actions and policies are perceived as supportive for PSWs. Semi-structured qualitative interviews were conducted with 25 PSWs actively working in the recovery field in South Carolina. Participants were asked “How does your employer or organization support you in the work that you do?” and “How could your organization support you better?” Interviews were recorded and transcribed verbatim. A team-based, rapid qualitative analysis approach was used to analyze data. Themes were identified and mapped onto existing guidelines for incorporation of PSWs into addiction medicine settings. PSWs reported that organizations can foster organizational support for PSWs through valuing PSWs as people with lived experiences. PSWs noted important supports such as living wages, insurance, job-related resources, and high-quality training and supervision. In addition, PSWs described how autonomy and reduced workload enable PSWs to optimize their time. Implications for organizations employing PSWs and recommended best practices are discussed.

## Introduction

The substance use crisis in the USA persists as a public health threat, with 25% of US residents age ≥ 12 years reporting using illicit substances and 17% meeting criteria for a substance use disorder (SUD).^1^ Overdose deaths have risen dramatically over the past two decades, with 105,000 drug overdose deaths reported in the USA in 2023 alone—primarily fueled by the ongoing opioid epidemic.^2,3^ The opioid crisis began with mass overprescription of opioids in the 1990s, followed by a rise in heroin use around 2010 and an increase in synthetic opioid use around 2013.^4,5^ The crisis is currently in its “fourth wave” which is marked by polysubstance use, with synthetic opioids, such as fentanyl and its analogues, frequently being laced into and/or used together with other substances, predominantly stimulants, presenting unique challenges and a greater risk of fatal overdose.^4,6^ Thus, timely efforts to address the crisis are crucial.

Peer support workers (PSWs) are individuals with lived experience with recovery from either SUD or mental illness who work in their respective fields to aid individuals’ treatment and/or recovery. Within the context of the SUD recovery field, PSWs are individuals with experience living with SUD and recovery, and use their knowledge of the processes, systems, and challenges impacting recovery to professionally support others with SUD with their recovery.^7^ Qualitative research highlights the many roles PSWs may fill that are enhanced by their “expertise-by-experience,”^8^ including fostering realistic and grounded hope for recovery, rendering compassionate clinical care, advocating for their clients, sharing personal recovery stories, building long-lasting relationships, and repairing distrustful relationships between people living with SUDs and healthcare systems that frequently approach these clients with stigma.^9^

PSWs are increasingly being integrated into the mental health treatment and SUD recovery fields.^7^ The US Substance Abuse and Mental Health Services Administration (SAMHSA) has established a national certification model for PSWs, though state-by-state variation exists in certification processes and training requirements and some PSWs do not obtain certification.^10,11^ PSWs’ roles and responsibilities widely vary, but are united by the common function of leveraging their lived experiences to enhance and fill gaps in care systems.^7,12,13^ Indeed, PSWs improve individual’s SUD-related outcomes (i.e., reductions in return to use, increases in treatment satisfaction, improved provider relationships).^14,15^ PSWs also report that their work contributes to their own personal growth and wellness.^16^

Understanding ways organizations can support PSWs is crucial given the expansion of the peer support workforce in the SUD recovery field. The social-ecological model of health understands individuals’ well-being as intertwined with a network of systems.^17,18^ As such, organizational policies, procedures, and climates impact PSW well-being. According to organizational support theory, perceived organizational support (POS), or employees’ perceptions of the degree to which their organization values and cares about their well-being, predicts workplace outcomes.^19^

Greater POS is associated with a host of positive work-related outcomes including higher job self-efficacy and satisfaction, better perceived work-life balances, fewer work-family conflicts, lower stress and burnout, and greater employee well-being.^19,20^ POS has been found to be predicted by organizational policies and procedures, including perceived fairness in treatment, human resources practices (i.e., job security, developmental opportunities, role flexibility), perceived respect for employees, and the amount and quality of resources available to employees.^19,20^ These relationships have been established with PSWs in the mental health treatment field, where POS is associated with higher job satisfaction.^21^

Given the crucial role that POS plays in PSW well-being as well as their job effectiveness, SAMHSA developed a set of recommendations for organizations aiming to integrate PSWs into addiction medicine settings.^10^ These recommendations relate to workplace climate, human resources policies and procedures, training and supervision opportunities, and sustainability of efforts. Because research to date has been limited with PSWs working in SUD treatment settings, these recommendations were primarily formed based on research with PSWs employed in mental health treatment settings.

SAMHSA recommends creating a workplace climate in which PSWs are valued as a key component of the organization, ensuring that non-peer staff members receive training on and demonstrate respect for the PSW role.^10^ Existing literature has shown that organizational culture and climate predict PSWs’ well-being in mental health settings, with PSWs feeling supported when colleagues convey respect and value for their roles.^22–24^ Likewise, one large study that included PSWs in both the mental health treatment and SUD recovery fields found that organizational commitments to authentic peer support, a culture of respect and collaboration, and coworker education on roles were crucial for POS.^25^

SAMHSA has several recommendations related to human resources policies and practices with PSWs, including assessing readiness prior to employing PSWs, hiring a sufficient number of PSWs, providing adequate career pathways, having clear job descriptions, offering equitable compensation compared to other employees, and utilizing strategic recruiting and interviewing procedures.^10^ Organizations in the mental health field that include continual partnership with PSWs in defining their roles and/or include PSWs in major leadership roles are perceived as more supportive to PSWs.^26,27^ Furthermore, research suggests that organizations can support PSWs through offering training opportunities, relevant work resources (e.g., computer, phone, internet access), upward mobility opportunities, and well-being resources.^23,24,27^ One study that included both PSWs in mental health and the SUD recovery field found that including peers in leadership positions was crucial for perceived support.^25^

In terms of PSW training and supervision, SAMHSA recommends initial orientations for PSWs to learn about their organizations and colleagues, ongoing training opportunities, and provision of regular supervision, ideally from a peer with lived experience.^10^ Provision of both formal and informal supervision from individuals with lived experience has been shown to enhance POS for PSWs.^25–27^ Finally, when discussing sustainability, SAMHSA emphasizes the importance of identifying sustainable funding for PSW services and utilizing data to demonstrate their value.^10^

Despite the breadth of research on factors related to POS among PSWs in the mental health treatment field and existing recommendations from SAMHSA, limited research has examined organizational support for PSWs working in the SUD recovery field. Existing research has typically engaged PSWs employed across both fields (i.e., mental health treatment, SUD treatment). Indeed, a recent scoping review indicated several key differences in responsibilities that may impact job-related needs.^28^ PSWs in the mental health treatment field often implement manualized-based treatments, while PSWs in SUD treatment are much more likely to link peers to services and aid in treatment initiation. PSWs in SUD treatment are also more likely to serve as sources of emotional support.^28^ The differing roles may require different skillsets and place a different emotional toll on the PSW. Moreover, PSWs have been integrated within the mental health treatment field for longer than in the SUD recovery field. As such, PSWs in the SUD recovery field face unique challenges in their work, such as SUD-related stigma, navigating multiple pathways to recovery, and trauma exposure associated with the overdose of peers.^25,29,30^ Additional research that focuses exclusively on PSWs working in the SUD recovery field is needed to ensure guidelines and organizational recommendations are tailored to this setting.

The present study aimed to use a qualitative approach, centering the voices of PSWs in the SUD recovery field, to determine what organizational policies and procedures foster POS for PSWs in the SUD recovery field. Although it was anticipated that PSWs in the SUD recovery field may have unique needs compared with other PSWs, no a priori hypotheses were made.

## Methods

### Study design

Semi-structured individual interviews were conducted virtually to elicit PSWs’ perspectives on their roles and existing and needed organizational support. The Standards for Reporting Qualitative Research were used as a guide for reporting.^31^ A brief questionnaire was used to gather demographic and employment data. To help promote accurate interpretation of the findings, a PSW currently working in the recovery field was engaged as a paid consultant to review all study materials prior to data collection and to member-check findings following data analysis. Results from other portions of this study have been published elsewhere.^13,32,33^

The researchers used a constructivist approach, recognizing that results are constructed within contexts and systems. Because researchers’ positionalities and lived experiences naturally influence their interpretation of data, the positionalities of the interview and analysis team are described (i.e., first through sixth authors) for transparency. The researchers identified as a cisgender White woman from a small midwestern town, a White American genderqueer person from a small town in rural Central Appalachia, a cisgender biracial woman of Chinese and German heritage who was born and raised in a suburban town in the Southeast United States, a cisgender woman, first-generation college student, and daughter of low-income Chinese immigrants from a large metropolitan city, a biracial Filipino-Polish American and cisgender woman, and a cisgender White woman from a suburban midwestern town. At the time of data collection and analysis, all analysis team members were doctoral psychology students completing a fellowship in SUD prevention and treatment who each had experience providing both mental health and consultative services to individuals with SUDs. At the time of data collection and analysis, none of the analysis team had worked as a PSW in the SUD recovery field. These identities and the lived experiences that flow from them naturally shape the analytical team’s understanding of what participants shared within interviews. To limit potential biases in the authors’ understanding of participant responses, the authors used a team-based analytical approach and employed a PSW consultant, as previously described.

### Procedures

All study procedures were approved by the University of South Carolina Institutional Review Board (IRB). A recruitment flyer was distributed via email to SUD recovery-oriented organizations and agencies across South Carolina. To be eligible, participants were required to (1) be ≥ 18 years of age, (2) have been a PSW for more than three months, and (3) be able to complete a survey and interview in English. PSWs were required to have been a PSW for more than three months to ensure that they had some experience in the role prior to participating in the study.

All participants provided verbal consent for participation. With IRB approval, written consent was not obtained as this would create unique identifiable information. Participants first completed a brief web-based survey. Next, they participated in a virtual individual qualitative interview. Individual interviews were conducted using a secure video platform and lasted 39 to 104 min, with a mean interview time of 59.4 (*SD* = 16.8) minutes. Six trained interviewers (i.e., first through sixth author) conducted the interviews in an individual fashion, with each member conducting between three and five interviews. Participants were compensated with a $50 gift card for completing the survey and interview. The recorded interviews were transcribed verbatim and cross-checked for accuracy. Interview transcripts were de-identified using pseudonyms that were either selected by participants if desired or randomly generated by the research team. The interview team met regularly to reflect on field notes and to discuss any challenges in conducting interviews.

### Measures

The semi-structured interview guide was created by the study team based on prior literature related to PSWs and the social-ecological model of health.^17,18^ The interview guide was reviewed and edited by the PSW consultant to the project. This study focused on organizational supports for PSWs, which were addressed through the following questions: “How does your employer/organization support you in the work that you do?” and “How could your organization support you better?” Participants also completed a brief questionnaire reporting sociodemographic and employment characteristics.

### Participants

The target sample size was set to 25 participants based on budgetary considerations and to ensure sufficient interviews to reach data saturation. A total of 25 PSWs completed the study. Thirty-nine PSWs completed the interest survey, all of whom met eligibility requirements. Eight of these individuals did not respond to contact attempts to schedule participation and were removed from the study. The final six individuals to complete the interest survey were notified that study enrollment was full and thanked for their interest.

### Data analysis

A team-based, rapid qualitative analysis was used for analysis due to the a priori developed research questions and to allow for rapid dissemination of findings given the changing landscape of the SUD crisis in the US.^34,35^ Rapid qualitative analysis has been validated as comparable to other methods such as thematic analysis while yielding quicker results.^36,37^ Rapid qualitative analysis involves a process of creating summaries of responses to each question asked within the interview and then entering responses into a coding matrix for interpretation.^34,35^

The following steps were used for rapid qualitative analysis. The interview guide and field notes were used to develop domain names which were placed in a summary template. To test the summary template, the analytical team listened to the same randomly selected interview recording while reading the transcript, inserting relevant detailed notes and illustrative quotes from the interviews into the summary template. The team then met to ensure consistency in coding practices and modify domain names as needed and ensure coding was occurring at similar levels among the six coders. Next, the remaining transcripts were divided among the analysis team for analysis such that one team member analyzed each transcript. After all interviews were coded, the summary template results were entered into a coding matrix using Microsoft Excel. The coding team met to review the extracted data and identified substantive themes. The study consultant cross-validated themes after consensus was established by the team. After themes and representative quotations were identified for each theme, the first and second authors compared themes with SAMHSA guidelines for integrating PSW into addiction medicine settings to identify areas of commonality and discrepancy.^10^

## Results

This study aimed to determine what organizational policies and procedures foster POS for PSWs working in the SUD recovery field. Twenty-five PSWs who provided services to people with SUDs in South Carolina participated. All participants were certified in their role as PSWs. The mean age was 49.3 years (*SD* = 12.9). Most identified as cisgender women (*n* = 16; 64%) and White (*n* = 20; 80%). Most participants were paid full-time employees as PSWs at the time of the study (*n* = 21; 84%). Participants reported having worked an average of 3.2 years (*SD* = 2.3) as a PSW. Full participant sociodemographic data is provided in Table 1.

**Table 1:** Sociodemographic information of participants

	*N* (%)	Mean (SD)
Age in years		49.3 (12.9)
Gender		
Cisgender woman	16 (64)	
Cisgender man	9 (36)	
Race		
Black or African American	3 (12)	
White	20 (80)	
White and Native American/Alaska Native	2 (8)	
Highest level of education		
High school/GED	2 (8)	
Some college	8 (32)	
Associate degree	6 (24)	
Bachelor’s degree	6 (24)	
Master’s degree	1 (4)	
Doctoral or professional degree	2 (8)	
Employment		
Paid full-time employee	21 (84)	
Paid part-time employee	3 (12)	
Part-time volunteer	1 (4)	
Years in personal recovery		12.5 (9.6)
Years working as PSW		3.2 (2.3)

PSWs shared ways in which they felt supported by the organizations, as well as ways in which they desired additional support. Key themes were arranged according to SAMHSA’s recommendations for integrating PSWs into addiction medicine settings and encompassed themes related to (1) workplace climate, (2) human resources policies and practices, (3) training and supervision provision, and (4) sustainability and funding. Table 2 contains key themes and exemplar quotations mapped onto SAMHSA’s recommendations. All themes identified from analyses mapped onto these four categories from SAMHSA. A discussion of key themes for each of these broad topics follows. Participants identified both *existing perceived supports* (i.e., supports they currently received) and *participants’ recommendations* (i.e., supports that were desired but not currently available to participants). Key quotations are shared throughout the results section credited to *pseudonyms* to protect participant privacy.

**Table 2:** Emergent themes compared with SAMHSA’s PSW integration recommendations^10^

	SAMHSA recommendations	Theme	Exemplar quotes	Novel or discrepant findings
Workplace climate	**Shifting the organizational culture:** PSWs should be viewed as a key ingredient to SUD care, not an add-on service. Organization administrators should communicate to nonpeer staff the value of PSWs. **Ensuring respect of PSW role:** Staff should be committed to recovery principles and avoid stigmatizing language or actions. PSWs should be included in organizational activities and functions, including informal gatherings.	PSW felt support when organizations value PSWs as a person, a person with knowledge and lived experience, and as a team member.	I feel very respected…I’m treated like I’m a professional. You know, even though I don’t have a bunch of letters, you know, attached to my name… my experience and my knowledge is valued and respected. – Bernard, 37-year-old cisgender man I don’t have any kind of like..I don’t have a bachelors [degree]. I don’t have an associates [degree]. I’m in the rooms with people with masters degrees and we’re tossing up ideas because they’re valuing my lived experience. I mean that's priceless. – Todd, 45-year-old cisgender man	PSWs emphasized being valued for their lived experience, particularly among individuals who have higher levels of education than them. Organizations should ensure PSWs have an equal voice in discussions and decision making as other staff.
	**Championing PSWs:** Organization administrations should champion PSWs, identifying allies among non-peer staff. Non-peer staff should understand the responsibilities of PSWs. **Training Non-Peer Staff:** Nonpeer staff should be trained on the roles of PSWs as well as what learning what training PSWs have received.	Organizations should promote understanding of PSW role/responsibilities to increase understanding/value of PSW.	Educating from the top at each because oftentimes, the administrators for these, they don’t know what these peer what peers are intended for. They don’t know their value. And if they don’t know their value, they're not going to be able to be implemented well. – Todd, 45-year-old cisgender man	PSWs emphasized the importance of educating not only non-peer colleagues, but also non-peer leadership on their role and value.
Human resources policies and practices	**Assessing readiness: **Prior to hiring PSWs, organizations should ensure they are ready to incorporate PSW into their organization. This can be assessed through organizational values, policies and practices, staff knowledge and attitudes, and supervision and support opportunities.	Not discussed	NA	NA
	**Setting hiring objectives for direct employees: **PSWs ideally should not be hired in isolation. In addition, when hiring PSWs, organizations should attend to diversity, hiring PSWs who have similar experiences as their clients. **Develop a community of PSWs:** PSWs should have access to and engage with peer-only staff meetings, either through their organization or with others beyond their organization if insufficient PSW staff is available. Provide a career pathway: Career ladders and opportunities for advancement are critical for PSWs	Organizations should hire more PSWs and have opportunities for different pay scales and responsibilities.	The brutally honest answer to that is they could hire another peer support specialist. – Bob, 42-year-old cisgender man I guess with county agencies we you know there are so many people to see and that becomes a kind of pressure in itself and so you can’t take things slowly, and you know when you start to feel like you know, even jotting down your notes from a session is getting in your way of actually meeting with people. You know, it’s—we need to slow down – Akiva, 52-year-old cisgender man It should be already established the tier of peer support [you are]. [If] you coming you had three years opposed to someone that got a year or two or three months, you should be able to teach or you know, show, or coach them the ropes – Stella, 62-year-old cisgender woman	PSWs shared that hiring enough PSWs is important to ensure that they are not overburdened with work, as well as not being isolated. PSWs highlighted the importance of upward mobility, not only in payscale but also in job responsibilities.
	**Setting a compensation package:** PSWs should be paid a fair wage with benefits. Benefits may include health insurance, paid time off, sick leave, employee assistance programs. Mileage reimbursements for travel in personal vehicles are recommended. Full-time positions should be offered, though ramifications related to public benefit access should be discussed with PSWs. Flexible work schedules may be useful.	PSWs valued being paid a livable wage, getting good insurance, having employee assistance program (EAP services), affordable therapy, and paid time off PSWs described wanting more mental health supports, through various means (e.g., therapy, anonymous line, more mental health days).	Our employer has a great benefits package. And they paid the majority of the cost. They pay the program for you can get therapy, which I like. – Tinkerbell, 59-year-old cisgender woman Something to sort of give people the outlet…you know, actually being able to say I need a mental health day. – Akiva, 52-year-old cisgender man	Mental health resources are particularly important benefits for PSWs, in addition to other standard benefits.
	**Writing a job description:** Job descriptions should clearly establish the duties of PSWs as well as how PSWs interact with other staff positions.	PSW felt support when organizations allowed PSWs to have flexibility in their roles and responsibilities, as well as creativity and autonomy. Organizations should reduce logistical/procedural burden on PSWs when receiving/building up new programs/grants.	I am a very independent person and those organizations have allowed me to, like, embrace that independence.– Britney, 24-year-old cisgender woman I see peers getting substituted for other areas of work, and usually they’re more, they’re more menial jobs. – Todd, 45-year-old cisgender man	PSWs valued having input into their job descriptions and roles.
	**Recruiting:** Organizations can partner with state credentialing organizations to advertise positions.	Not discussed	NA	NA
	**Interviewing:** Existing PSWs should be consulted in the development of interview questions and hiring process. Some example interview questions may focus on how individuals respond to complicated scenarios.	Not discussed	NA	NA
Training and supervision provision	**Preparing an orientation for PSWs**: PSWs should be given a thorough orientation to the organization, beyond their training as a PSW. This may involve learning about the roles of various staff members at their organization.	Not discussed	NA	NA
	**Promote ongoing training**: PSWs should have access to formal training, on the job coaching, and continuing education in their role. Organizations can support this through providing paid time to attend trainings, covering the cost of continuing education, and supporting experienced PSWs training less experienced PSWs.	PSWs feel supported when organizations encourage, identify, provide, allow, and/or cover PSWs to partake in trainings.	Whenever there is a training that comes up that someone wants to take, most times we find a way to fund it. – April, 56-year-old cisgender woman	In addition to providing financial coverage of trainings, organizations can support PSWs by identifying training opportunities and encouraging attendance.
	**Arranging for supervision for PSWs**: PSWs should receive supervision from someone with a recovery outlook, ideally with experience working as a peer.	PSWs feel supported when organizations provided supervision to PSWs, which was a space for PSWs to receive support/check in PSWs want more supportive peer support supervision, particularly from people with lived experiences, and additional training opportunities.	Direct supervision weekly. And it is the first, at the first day of every week and sit down with my supervisor to where we can go over things and talk about things. That is essential. – Bob, 42-year-old cisgender man Peer support supervision. Like an individualized like a peer support who is actually, you know, who actually knows exactly what peer supports do like my supervisor is amazing, but she's been learning kind of like as we go. I think it would be helpful if, if there was a specialist supervision for a peer support. – Lucy, 42-year-old cisgender woman They [clinically trained supervisors] don't really know how to supervise because the roles are not the same. The ethics are not the same…And and so consequently I mean and you know and supervision is hard on the best of days with the where the clinician is working about on the same set of rules that you are but there’s a lot of gray in peer support – Akiva, 52-year-old cisgender man	PSWs emphasize that provision of supervision from individuals with lived experience is paramount as the roles and challenges differ from that of mental health providers.
Sustainability and funding	**Identifying funding:** Organizations should understand funding opportunities available for PSW, including Medicaid and other insurance reimbursement policies and available grants for PSW. **Sustainable funding:** Organizations should ensure consistent funding is available for PSWs to avoid discontinuing services. Organizations can look for funding through insurance reimbursements and grant programs.	Organizations should seek more funding/money, for a variety of reasons.	Find funding and increase company monetary compensation…not only could we pay ourselves completely, we could also implement additional programs. – April, 56-year-old cisgender woman I’ve seen job postings for $12.00 an hour. I don’t know how somebody you know people could survive on that alone, much less with a family.—Todd, 45-year-old cisgender man	PSWs report funding should be sought to ensure they are being paid living wages as well as funding initiatives and programming.
	PSW evaluation, marketing, and advocacy: Organizations should collect and share information regarding PSW effectiveness with funders, key stakeholders, and communities at large.	Not discussed	NA	NA

### Workplace climate

*Existing perceived support: PSWs felt supported when recovery organizations valued PSWs as a person with knowledge and lived experience and treated them as a respected team member.* PSWs reported appreciating when their contributions and expertise, as someone with lived experience, were valued, as well as when they were given an equal voice as those with higher education or professional degrees. As JB, a 61-year-old White cisgender man stated, supportive organizations “value what we bring to the table.” In addition, PSWs noted valuing when their coworkers, supervisors, and leaders demonstrated a genuine concern for them, checking in on their well-being, and encouraging self-care.

*Participants’ recommendation: Organizations should promote understanding of PSW role/responsibilities to increase understanding of and value for PSWs.* PSWs voiced a desire to have staff and leadership better understand the unique role and value of PSWs in the recovery space. They noted the importance of providing education and training on these topics to non-PSW staff. They also shared that some coworkers may need to explore their biases related to SUDs, recovery processes, and the role and value of peer support as a part of this process. As Sylvia Jane, a 45-year-old cisgender woman, said “[My] organization could support me…by properly training the entire staff on what peer support is [and] how peer support is valuable.”

### Human resources policies and practices

*Participant’s recommendation: Organizations should hire more PSWs and have opportunities for different pay scales and responsibilities.* Cassidy, a 65-year-old Black cisgender woman, shared that “another peer support person would be nice.” Many PSWs described being the only PSW at their organization which was isolating and prevented them from sharing their workload. They indicated that additional PSW colleagues would enable them to take time off when needed, knowing that another PSW would be able to serve their clients in need. In addition, PSWs shared that having opportunities for upward mobility, including different job responsibilities and higher pay based on experience level, would be beneficial.

*Existing perceived support: PSWs valued being paid a livable wage, having good insurance, being provided with an employee assistance program (EAP), having access to affordable therapy, and receiving paid time off.* Todd, a 45-year-old White cisgender man, described that offering these benefits showed that his organization “believe[d] in the work that you’re [PSWs are] doing.” As Todd further reflected on his organization compared with others, he shared “I’ve seen job postings for $12.00 an hour. I don’t know how somebody, you know, people could survive on that alone, much less with a family,” highlighting the importance of being paid a living wage. Other PSWs shared how organizations they had worked with in the past had not provided sufficient salaries, and they needed to work part-time in addition to their PSW role to cover basic living costs. PSWs also shared the benefits of having access to either insurance that covered therapy or having their own therapy covered by their employer directly.

*Participants’ recommendation: PSWs described wanting more mental health supports through various means.* PSWs desired to have the opportunity to take more days off for their mental health. Related to therapy, some reported they did not have access to therapy or had a large deductible that made therapy visits very difficult. Others reported it would be helpful to have access to counseling within their organization or an anonymous support line specifically for PSWs. April, a 56-year-old cisgender woman, shared that because her organization was small, they were unable to provide mental health services for PSWs “those [mental health days and covered cost of individual therapy sessions] are very good and…we aspire to build those.”

*Existing perceived support: PSWs felt supported when their organizations allowed PSWs to have flexibility in their roles and responsibilities, as well as creativity and autonomy.* PSWs described the value of having flexibility in defining their roles and having autonomy to create and lead initiatives. Isaiah, a 77-year-old White cisgender man, shared that it was beneficial when organizations “give us our lead” to pursue new programming or project ideas. PSWs also shared it was helpful when they were given the ability to use funding in a way that was useful to them and had the opportunity to delegate to others. Finally, they shared the benefits of flexibility in their schedules, enabling them to meet their clients’ needs while also supporting their own recovery through attending mutual help groups when needed.

*Participants’ recommendation: Organizations should reduce logistical/procedural burden on PSWs when receiving/building up new programs/grants.* PSWs frequently felt “volun-told” (i.e., told to volunteer without a perceived option to decline) to perform overwhelming or complex procedural tasks. They noted it would be beneficial to limit the procedural and logistic burden for PSWs so they were able to focus their time on tasks that could uniquely be completed by PSWs compared with office staff.

### Training and supervision provision

*Existing perceived support: PSWs felt supported when their organizations encouraged, identified, provided, allowed, and/or covered PSWs to partake in trainings.* PSWs appreciated it when their organizations shared relevant training opportunities with them and encouraged them to attend. Of note, many PSWs highlighted the value of their organization, covering both the cost of trainings and paying them for their time attending these trainings. For example, Glen, a 41-year-old cisgender man, shared his organization had “gone above and beyond… I don’t pay for any of those [trainings] out of pocket. They actually pay me to go to the trainings.”

*Existing perceived support: PSWs felt supported when their organizations provided supervision to PSWs, which was a space for PSWs to receive support/check in*. PSWs emphasized the value of this supervision in creating a space for them to receive support and to ensure they are not overworking. Notably, while some PSWs shared the value of this supervision, others noted they felt they were not receiving sufficient supervision.

*Participants’ recommendation: PSWs wanted more supportive peer support supervision, particularly from people with lived experiences, and additional training opportunities.* PSWs noted that they would like peer support specific supervision, with supervision by “seasoned PSWs” rather than clinicians, given the different roles of the professions. Akiva, a 52-year-old cisgender man shared that “there was not a whole lot of supervision…you feel like you’re kind of cut off.” PSWs also highlighted the value of having additional opportunities for ongoing training, particularly in the realm of self-care, professional boundaries and self-disclosure.

### Sustainability and funding

*Participants’ recommendation: Organizations should seek more funding/money, for a variety of reasons.* PSWs noted that funds were unavailable or tight. As Mira, a 38-year-old White woman, said, PSWs need “More money. I don’t wanna cloud that with anything else. More money.” PSWs noted that additional funds would not only enable funding for more equitable compensation for PSWs but also could fund implementation of more high-impact PWS-led programs. In addition, PSWs desired financial leniency to make purchases related to their work, such as purchasing business cards.

## Discussion

PSWs are a key workforce supporting the US response to the SUD crisis, yet limited research has examined ways that recovery-oriented organizations and agencies can better support PSWs in their roles. The present study, guided by organizational support theory, aimed to understand ways in which organizations can meet the needs of their PSW workforce. PSWs identified existing and needed supports related to their workplace climate, human resources practices and policies, training and supervision provision, and sustainability and funding of their role.

### Workplace climate

With respect to workplace climate, PSWs noted that it was important to feel like and be treated as an equally valued member on interdisciplinary service teams, which may be facilitated in part by promoting a better understanding of PSW roles, responsibilities, and value among other professionals. This finding is consistent with organizational support theory, prior research from the mental health field, and existing SAMHSA guidelines.^10,19,20,22–25,38^ PSWs described how feeling valued by their interdisciplinary teammates was very important, especially given that they often had less formal education than their interdisciplinary team coworkers. Of note, while SAMHSA guidelines emphasize the importance of ensuring peer support is seen as a “key ingredient” of care, participants in the present study further emphasized feeling valued when their lived experiences were valued as a form of expertise. These themes underscore the challenges PSWs may face working in service settings that have historically prioritized formal education and certain credentials as the primary indicators of expertise.

Despite SAMHSA recommendations for creating a supportive workplace, many PSWs reported a lack of support.^10^ Consciously or not, biases among other professionals may marginalize the lived experiences and unique insights that PSWs bring to the table, resulting in their contributions being viewed as less legitimate. Trainings focused on challenging these misconceptions may be helpful in elevating the role of PSWs. Such trainings might address institutional biases through building awareness of biases, providing concrete examples of how PSWs in the SUD recovery field leverage their lived experience, and sharing research evidence on the effectiveness of PSW work. Trainings related to the value and role of PSWs should be routine, ongoing, and provided to staff across employment levels—including leadership, supervisors, and colleagues of PSWs. Notably, while SAMHSA recommendations emphasize this training for PSWs’ colleagues, PSWs in the present study identified the importance of organizational leadership having continual training on the role and value of PSWs.^10^ Leadership attitudes and behaviors can have a trickle-down effect, signaling values to the organization. Indeed, inclusive leadership is paramount for inclusive work settings.^39^ The authors echo and extend calls for trainings on the value of PSWs in the SUD recovery field, noting the importance of all organizational members receiving such training.^10,25^

Although frequently recommended, research suggests that workplace bias trainings, particularly one-off trainings, tend to have minimal and/or time-limited effects on actual behavior.^40,41^ However, bias trainings that are ongoing, implemented alongside other inclusion approaches, and focused on applying bias reduction strategies are more effective.^40,42^ In addition, contact-based and collaborative strategies can be useful to increase acceptance and inclusion in the workplace.^41^ As trainings and other procedures to increase acceptance of PSWs are implemented, research should be conducted to evaluate the effectiveness of such approaches.

### Human resources policies and practices

Regarding human resources policies and practices, the present study highlights the importance of hiring enough PSWs to promote manageable workloads, providing equitable compensation, and creating appropriate job descriptions. Ensuring sufficient PSWs are employed relative to workloads is crucial to ensure individual workloads are manageable.^10^ Participants discussed feeling overburdened and voiced a desire to ensure sufficient PSWs were hired to share workloads. Moreover, by employing multiple PSWs, organizations may facilitate social support between PSWs, further supporting PSW’s work and well-being.^10^ Related to individual workloads, overburdened PSWs are at risk for negative psychosocial outcomes as well as greater turnover.^43^ Thus, for sustainability of PSWs caseloads must be managed. Beyond the impact of individual workload, many PSWs reported being the sole PSW in their organization, which can be isolating and may contribute to feeling like one does not belong. In organizations where few PSWs are employed, SAMHSA recommends organizations should ensure they are providing opportunity and funding for PSWs to convene with PSWs at other organizations for support.^10^

Clear job descriptions and expectations for what roles and skills (beyond those of expertise by virtue of experience) are to be performed by PSWs are likely to be beneficial. PSWs as professionals are relatively new additions to care teams, and many may not be given clear guidance on the details of their role, their function within a care team, or the benefits their job entitles them. Additionally, greater communication about PSWs’ job expectations may serve to lessen potential workplace stigma, or treatment of PSWs as “less-than,” by a care team that is otherwise ignorant to the value provided by PSWs. Greater openness about workplace expectations among PSWs and care teams broadly will not only support good fit between PSW applicants and organizations and communicate an organizational value for the knowledge they bring, but will also facilitate greater cohesion between PSWs and the continuum of care.

PSWs in the present study noted the importance of being paid a living wage to complete their jobs to the best of their abilities, which is consistent with current recommendations.^10,38^ Despite clear recommendations for pay parity, salaries for PSWs remain low, with full-time PSWs making less than a living wage for a single person in 19 states and less than a living wage for a single adult with one child in all states.^44^ Provision of a living wage is not only important for PSW retention and quality of life, but it also conveys organizational values. PSWs must be paid equitable, living wages, to sustain their work in addressing the ongoing SUD crisis.

Relatedly, PSWs highlighted the importance of mental health resource provision including access to affordable mental health treatment and the opportunity for taking time off for mental health. Prior research has noted the importance of mental health supports for PSW general well-being.^23,27^ PSWs not only are managing their own recovery but also are routinely exposed to potentially traumatic or triggering experiences with their clients, making mental health service access critical. Organizations should support their PSWs through providing benefits packages that cover mental health and substance use disorder services at an affordable rate. Moreover, organizations can provide further support through covering costs of co-pays or other mental health-related costs for PSWs.

PSWs shared they valued having flexibility and autonomy in their roles and responsibilities, such as having opportunities to initiate programming and using their individual skillsets to address challenges when they occurred. Concurrently, PSWs voiced frustration when they were sometimes asked to complete “menial” tasks such as scheduling appointments, making copies, or completing paperwork. Whereas flexibility allowed PSWs to exercise their expertise from their lived experiences, being delegated administrative tasks conveyed to PSWs that their contributions were limited, reinforcing a workplace climate in which they felt devalued. This assignment of tasks can stem from a negative workplace climate as well as poor role definitions. SAMHSA recommends having clearly described duties for this reason.^10^

PSWs likely benefit from clear job descriptions that explicitly allow flexibility and autonomy in selecting duties based on their self-perceived individual strengths and interests. For example, while one PSW shared that they enjoyed lending their expertise to writing grants, another PSW may prefer to focus on work with individual clients or on programming. Allowing for continual negotiation of the specific job responsibilities for PSWs with an emphasis on PSW input may improve POS as well as help ensure the resource of PSWs’ time is used appropriately and effectively.^27^ Creating flexibility in roles may be a way to increase the number of PSWs in critical leadership positions, which is an important predictor of POS.^19,20,26^ Notably, participants in the present study voiced a desire for opportunities for upward mobility – not simply for increasing salaries, but also to gain additional job responsibilities.

### Supervision and training

Consistent with prior research and SAMHSA’s recommendations, PSWs highlighted the importance of receipt of high-quality supervision from an individual with lived experience as a PSW.^10,25–27^ Notably, while SAMHSA’s guidelines indicate it is “ideal” for PSWs to be supervised by PSWs, they acknowledge this is not always feasible.^10^ However, PSWs felt they were receiving insufficient supervision when being supervised by mental health providers rather than PSWs due to differences in peer support work from mental health clinical work in roles, confidentiality limits, work-life boundaries, and expectations related to disclosure. Supervisors who are PSWs themselves could offer better guidance related to the role of PSWs and navigating challenges faced by PSWs that may not be faced by mental health clinicians.

Given the unique roles of PSW in the SUD recovery field (e.g., engaging in more care linkage and treatment initiation, emotional support role), the need for supervision from a fellow PSW may be particularly crucial for PSW in the SUD recovery field compared with mental health fields. Organizations in which supervision of PSWs by PSWs is not feasible should seek out opportunities for outside supervision of PSWs by PSWs.^10^ While clinically trained supervisors may be able to provide some guidance to PSWs, the inherent difference in responsibilities and experiences creates a need for supervision by PSWs themselves.

PSWs valued opportunities to engage in continuing education and training and appreciated when organizations shared these training opportunities and covered relevant costs, which is consistent with prior research in the mental health field.^23,24,27^ PSWs emphasized the value of their organizations providing recommendations on trainings to attend, which extends the recommendations from SAMHSA that are focused on funding training.^10^ In recommending trainings, organizations should be attuned to trainings that create opportunity for upward career mobility, as this is another key predictor of POS.^19,20^ While the implementation of local training opportunities may be challenging, particularly in rural areas, opportunities for remote and collaborative training opportunities may be more accessible. For example, the Extension for Community Healthcare Outcomes (ECHO) is a model that has been used to increase training opportunities for providers and PSWs within the SUD field and shows promise.^45^

### Sustainability

In terms of sustainability, in the present study the importance of funding was highlighted. PSWs highlighted the importance of hiring additional PSWs, increasing wages, and funding PSW-led initiatives. An additional factor increasing the importance of hiring more PSWs is the diversity of recovery experiences within the field of SUD recovery. Very little empirical information is available regarding whether PSWs are hired for their specific expertise based on prior use history (e.g., history of opioid use versus alcohol use), or are simply hired for their assumed knowledge based on recovery generally. Experiences of use and recovery are different based on substance—typically involving a disparate trajectory of initial use, health and social consequences of long-term or increasing use, and medical and behavioral supports required for recovery. Empirical research has not yet discussed whether, for example, the lived experiences of a PSW in recovery from opioid use translate to effective experience-as-expertise to help someone in early recovery from stimulant use. Additional study is needed to understand which lived experiences are most valuable for the different functions and roles that PSWs serve.

Offering responsive support to a robust workforce of PSWs with varied experiences is a consistent recommendation between SAMSHA guidelines and the thoughts of PSWs in this study. Beyond salary, participants also emphasized a desire for sustainable funding for peer-led initiatives and programming as well, an area that extends beyond SAMHSA guidelines. For these reasons, the authors echo recent calls for financial investment in the PSW role, not only in the individual salaries, but also in the number of PSWs employed and the discretionary funds available to them.^25^ By financially supporting the work of PSWs, organizations can reap the benefits associated with effective peer support work and better address the SUD crisis.

### Limitations and future directions

The present study has several limitations worth noting. First, the sample used a convenience sampling method, with participants volunteering based on a flyer that was distributed throughout the state of South Carolina. The individuals who selected to participate may differ in their job satisfaction and felt organizational support compared to those who did not participate. In addition, participants worked at a diversity of locations throughout the state and there was significant heterogeneity in their experiences of support as a result. While this allowed for a wide range of perspectives to be shared, there was significant variation in the perceived support from organizations. Future research may benefit from examining practices of organizations where PSWs feel particularly supported.

The present study demonstrated the ways in which organizational support theory operates for PSWs in the SUD recovery field through qualitative methods. Future research may examine these variables through quantitative methodologies, examining predictors and outcomes related to POS in PSWs. This future research would strengthen the field’s understanding of necessary organizational actions to support PSWs.

## Implications for Behavioral Health

PSWs working in the SUD recovery field play a vital role in addressing the nation’s ongoing opioid epidemic, an urgent public health crisis that demands immediate and multi-level intervention. Organizations can and should take several tangible steps to ensure they are supporting PSWs in their work. Additional funding mechanisms should be created to assist organizations in paying PSWs an equitable wage. Organizations employing PSWs and those working at such organizations should actively engage in efforts to create policies and practices that are tailored to support and show value for the unique role of PSWs, as well as to support the sustainability of PSW-led programming through tangible actions.

## Data Availability

To protect the confidentiality of our participants, the data from this study are not publicly available.
